# Supplementary material for: Mild myelin disruption elicits early alteration in behavior and proliferation in the subventricular zone
Source: eLife. 2018 Feb 13;7:e34783. doi: 10.7554/eLife.34783 (PMC5828668; doi:10.7554/eLife.34783)
Supplement: Transparent reporting form [file elife-34783-transrepform.docx]

***eLife’s* transparent reporting form**

We encourage authors to provide detailed information *within their submission* to facilitate the interpretation and replication of experiments. Authors can upload supporting documentation to indicate the use of appropriate reporting guidelines for health-related research (see [EQUATOR Network](http://www.equator-network.org/%20)), life science research (see the [BioSharing Information Resource](https://biosharing.org/)), or the [ARRIVE guidelines](http://www.plosbiology.org/article/info:doi/10.1371/journal.pbio.1000412) for reporting work involving animal research. Where applicable, authors should refer to any relevant reporting standards documents in this form.

If you have any questions, please consult our Journal Policies and/or contact us: [editorial@elifesciences.org](mailto:editorial@elifesciences.org).

**Sample-size estimation**

- You should state whether an appropriate sample size was computed when the study was being designed
- You should state the statistical method of sample size computation and any required assumptions
- If no explicit power analysis was used, you should describe how you decided what sample (replicate) size (number) to use

Please outline where this information can be found within the submission (e.g., sections or figure legends), or explain why this information doesn’t apply to your submission:

No statistical method was used to predetermine sample size. At least 3 mice/genotype were used to ensure adequate statistical power analysis. LFPs were analyzed from 64 units/genotype. Sample size was increased to at least 10 mice/genotype for behavioral experiments. Using G power these sample sizes were determined to be adequate given the effect size and study design^1^. Sample size is cited within the figure legends for all studies.

1.	Faul, F., Erdfelder, E., Lang, A. G. & Buchner, A. G* Power 3: A flexible statistical power analysis program for the social, behavioral, and biomedical sciences. *Behavior research methods* (2007).

**Replicates**

- You should report how often each experiment was performed
- You should include a definition of biological versus technical replication
- The data obtained should be provided and sufficient information should be provided to indicate the number of independent biological and/or technical replicates
- If you encountered any outliers, you should describe how these were handled
- Criteria for exclusion/inclusion of data should be clearly stated
- High-throughput sequence data should be uploaded before submission, with a private link for reviewers provided (these are available from both GEO and ArrayExpress)

Please outline where this information can be found within the submission (e.g., sections or figure legends), or explain why this information doesn’t apply to your submission:

Biological replicates are reflected in the number of mice used per experiment for histological analysis and behavioral experiments. Each LFP recording electrode unit was considered a biological replicate. To ensure adequate estimation of cellular numbers per mouse, histological analysis was repeated on at least 3 sections per mouse as technical replicates. Light sheet and confocal imaging were used as complementary techniques to quantify cell number and they were remarkably consistent. For behavioral experiments, one cohort of mice was tested at 9 months and another cohort of mice was tested at 3 months. The tests were performed in the same order and not repeated on any mouse. An additional cohort was utilized for olfactory behavior analysis. Outliers were not identified in any analysis and no data was excluded.

**Statistical reporting**

- Statistical analysis methods should be described and justified
- Raw data should be presented in figures whenever informative to do so (typically when N per group is less than 10)
- For each experiment, you should identify the statistical tests used, exact values of N, definitions of center, methods of multiple test correction, and dispersion and precision measures (e.g., mean, median, SD, SEM, confidence intervals; and, for the major substantive results, a measure of effect size (e.g., Pearson's r, Cohen's d)
- Report exact p-values wherever possible alongside the summary statistics and 95% confidence intervals. These should be reported for all key questions and not only when the p-value is less than 0.05.

Please outline where this information can be found within the submission (e.g., sections or figure legends), or explain why this information doesn’t apply to your submission:

Statistical analysis methods are located in the methods section. For histological analysis and behavioral experiments the data was determined to be parametric. For studies with greater than two comparisons, we utilized a multivariate ANOVA or repeated-measures ANOVA to determine differences between genotypes and different ages or brain regions, respectively. Post-hoc analysis was performed with Sidak’s multiple comparison test. A student’s T-Test was used to determine differences between two groups. The LFP analysis did not yield parametric data. To compare LFP power between genotypes, we utilized Mann-Whitney U-test with false discovery rate correction for multiple comparisons^2^. p-values are reported in the figure legends. P-values greater then p>0.01 are reported as exact values.

2.	Curran-Everett, D. Multiple comparisons: philosophies and illustrations. *American Journal of Physiology-Regulatory, Integrative and Comparative Physiology* **279,** R1–R8 (2000).

(For large datasets, or papers with a very large number of statistical tests, you may upload a single table file with tests, Ns, etc., with reference to sections in the manuscript.)

**Group allocation**

- Indicate how samples were allocated into experimental groups (in the case of clinical studies, please specify allocation to treatment method); if randomization was used, please also state if restricted randomization was applied
- Indicate if masking was used during group allocation, data collection and/or data analysis

Please outline where this information can be found within the submission (e.g., sections or figure legends), or explain why this information doesn’t apply to your submission:

Cohorts of mice were genotyped and caged with littermates of the same sex. The genotypes were blinded for experiments and only mouse numbers were used as identification throughout the experiments. For behavioral experiments the order of the genotypes was randomized. This is mentioned in text and methods.

**Additional data files (“source data”)**

- We encourage you to upload relevant additional data files, such as numerical data that are represented as a graph in a figure, or as a summary table
- Where provided, these should be in the most useful format, and they can be uploaded as “Source data” files linked to a main figure or table
- Include model definition files including the full list of parameters used
- Include code used for data analysis (e.g., R, MatLab)
- Avoid stating that data files are “available upon request”

Please indicate the figures or tables for which source data files have been provided:

Figure 1, Source data for MATLAB counts:

<https://github.com/elizabethagould/PLPeGFP_image_analysis>

Figure 6, Source data for Awake-behaving recordings:

<https://github.com/restrepd/drgMaster>
